# Vitamin D and its relationship to performance and health during a competitive period in elite women's basketball and volleyball players

**DOI:** 10.14814/phy2.70224

**Published:** 2025-02-04

**Authors:** Álvaro Miguel‐Ortega, Julio Calleja‐González, Juan Mielgo‐Ayuso

**Affiliations:** ^1^ Faculty of Education Alfonso X “The Wise” University (UAX) Madrid Spain; ^2^ International Doctoral School University of Murcia (UM) Murcia Spain; ^3^ Faculty of Education and Sport University of the Basque Country (UPV) Vitoria Spain; ^4^ Faculty of Kinesiology University of Zagreb Zagreb Croatia; ^5^ Faculty of Health Sciences University of Burgos (UBU) Burgos Spain

**Keywords:** female, indoor, performance, vitamin D

## Abstract

Vitamin D [25(OH)D] is a key nutrient, although its level is often low in the general population. To investigate the relationship between vitamin D levels and muscle performance, and to analyze how vitamin D changes during a 16‐week competitive season and its relationship to the performance tests performed. Participant characteristics: age 25.1 ± 4.7 years; height 1.8 ± 0.1 m, and body mass 73.9 ± 15.4 kg. Vitamin D levels (ng/mL) were at T1 (September): 33.7 ± 14.7 (*n* = 23), and at T2 (January): 26.1 ± 7.3 (*n* = 23). Over 16 weeks of competition, participants' blood was analyzed to determine their vitamin D levels. Their athletic abilities were evaluated through various tests: vertical jumps (standing jump and countermovement jump); 20‐m sprint without direction changes; and intermittent endurance test; the vitamin D level decreased from T1 to T2 by −22. 40% [*p* < 0.05] but performance improved in all tests performed (SJ: 4.57%; CMJ: 6.94%; VO_2max_: 4.99% [*p* < 0.05]; 20 m: −1.83%). There is a relationship between vitamin D levels and physical performance in female indoor athletes. The results suggest that increased training load may also negatively affect vitamin D levels in elite female indoor athletes.

## 1 INTRODUCTION

Vitamin D (vD), whose first biologically active metabolite is 25‐hydroxyvitamin D [25(OH)D] (calcidiol), an essential trace element, plays a vital role in physically active individuals (Yagüe et al., 2020), but is frequently deficient in the general population (McKenna & Kilbane, 2022). Defining vD deficiency is challenging (Spiro & Buttriss, 2014), with a concentration of less than 20 ng/mL (50 nmol/L) (Spiro & Buttriss, 2014) being considered relative insufficiency. Deficiency causes catabolic effects in muscle tissue, leading to muscle weakness (Dzik & Kaczor, 2019), decreased muscle mass (Walrand, 2016), and decreased physical and athletic performance (Dahlquist et al., 2015).

A recent study found a positive correlation between serum vD levels and jump height, speed and power (Koundourakis et al., 2014). In addition, the literature indicates that physical and athletic performance is seasonal, as athletes around the world must adapt to various weather‐related challenges, which affects their performance, more so in terms of vD activated by solar processes affected by the time of year, peaking when vD levels are highest and declining when levels fall (Cannell et al., 2009).

In sports performance, this deficiency is particularly pronounced due to a lack of exposure to sunlight (Yoon et al., 2021) and low intake of vD (Owens et al., 2015). Indeed, there is evidence that certain populations, such as athletes (Malczewska‐Lenczowska et al., 2018), have different vD requirements according to age and gender (McKenna & Kilbane, 2022). Thus, some findings indicate that women consume more vD than men (Hosseinzadeh et al., 2017), although women tend to consume fewer micronutrients than men (Thein‐Nissenbaum & Hammer, 2017). There may also be gender differences in blood levels of vD (Kader et al., 2019), so it is of great interest to establish an individual‐level criterion based on hormone levels (Schelling et al., 2015).

The literature indicates that athletes who play indoor sports are more likely to develop vD deficiency compared to those who are active outdoors (Aydın et al., 2019; Maruyama‐Nagao et al., 2016) due to less exposure to sunlight (such as basketball and volleyball players) (Bârsan et al., 2023).

Both basketball and volleyball (Lockie et al., 2020), direct‐confrontation team disciplines, require great technical specificity (Miguel‐Ortega et al., 2023), and both are intermittent high‐intensity activities (Espasa‐Labrador, 2023; Mielgo‐Ayuso et al., 2014). In recent years, the field of sports medicine has shown a growing interest in the impact of vD in such disciplines (Mielgo‐Ayuso et al., 2018), indicating that a significant number of indoor athletes are deficient in vD (Farrokhyar et al., 2015; Grieshober et al., 2018).

In terms of body composition, research has shown that this aspect plays a crucial role in performance in these disciplines (Calleja‐González et al., 2018; Mielgo‐Ayuso et al., 2015) because excess fat mass increases energy demands (Malá et al., 2010) by acting as a dead weight in antigravity activities (Reilly & Doran, 2003). However, increased musculoskeletal mass is considered a positive indicator of athletic performance, contributing to greater power output during exercise and increased size and strength at high dynamic and static loads (Mala et al., 2015).

To evaluate performance the tests to be performed are (i): squat jump (SJ) and countermovement jump (CMJ), since maintaining adequate vD values reduces muscle damage and post‐exercise inflammation and modifies calcium transport in muscle contraction (Caballero‐García et al., 2021); (ii) maximal oxygen consumption (VO_2max_) indirectly, because of its relationship of vD levels with cardiorespiratory capacity (Marawan et al., 2019); and (iii) the 20‐m sprint because there is a statistically significant association between vD levels and muscle power and strength (Marantes et al., 2011). These tests are standard goal tests to determine performance in these sports disciplines (Aschendorf et al., 2019; Neal et al., 2018; Ramirez‐Campillo et al., 2021; Villalon‐Gasch et al., 2022).

Overall, the study of DV and performance has predominantly focused on male athletes, despite the knowledge that sex differences may affect performance and recovery (Hunter et al., 2023). This is mainly due to a reluctance to work with female athletes due to hormonal variations associated with the menstrual cycle and oral contraceptive use, although it seems counterproductive to exclude a large part of the athletic population due to these factors (Engseth et al., 2022). And to the authors' knowledge, there are no studies that analyze this, so our study has a twofold aim. The primary, to investigate the possible relationship between vD levels and muscle performance, as indicated by squat jump (SJ) and countermovement jump (CMJ) (Markovic et al., 2004), maximal oxygen consumption (VO_2max_) and 20‐metre sprint performance at two different times during the competitive season in female indoor sports players (PFISPs).

The secondary was to examine the evolution of vD during the 16‐week seasonal transition period and its relationship to the above tests. We hypothesized that, in both experimental sessions, vD levels would correlate with the participants' jumping, sprinting, and aerobic ability. We also expected that the transition period between times would have a positive impact on vD concentration and so on athletic performance.

## 2 MATERIALS AND METHODS

### 2.1 Participants

Twenty‐three professional female indoor team sports players (*n* = 23), and members of two teams in volleyball (*n* = 11) and basketball (*n* = 12) were recruited for the study. All participants competed at elite level in their respective disciplines, the Iberdrola Women's Volleyball League, and the DIA Women's Basketball League 1. Participants in both sports followed a similar diet prescribed by their respective coaching staffs.

Mean values for age (years) ± SD, height (m) ± SD and body mass (kg) ± SD were age 25.1 ± 4.7, height 1.8 ± 0.1, and body mass 73.9 ± 15.4, respectively. The sample size (SS = 21.75) was decided using the G*Power package (version 3.1.9.2) with a finite population of 23 participants (*n* = 23). A confidence level of 95% (with a margin of error of 0.05) was assigned, giving the event the same probability of occurrence or non‐occurrence, resulting in a large effect size between the two groups (ES = 0.9) with a power of 80% (Abt et al., 2020).

On average, the volleyball players, over the week, spent an average of 19.5 h training, excluding matches. This training regime consisted of three double sessions focusing on technical and tactical aspects of 120 min in the morning, followed by 150 min of physical conditioning in the afternoon. These sessions took place on Mondays, Wednesdays, and Thursdays. In addition, there were 2 days with one 180‐min session each, Tuesday for physical conditioning and Friday for technical and tactical aspects. Saturdays were reserved for competitions, while Sundays were appointed as rest days. In basketball, a typical work week consisted of 22.5 h of practice, not including competitions. It consisted of three double sessions, with 150 min of technical and tactical work in the morning and 180 min of physical training in the afternoon on Monday, Wednesday, and Thursday. On Tuesday and Friday, there were two 180‐min sessions, with Tuesday focusing on both physical and technical‐tactical aspects, while on Friday the emphasis was on tactical elements only. Matches were scheduled for Saturday or Sunday, followed by a rest day.

The exclusion criteria were as follows: (a) participants had to be able to play and train without guarantees (i.e., no injuries); (b) none of the participants had to have injuries, allergies, or hormonal disorders during data collection; and (c) none of the participants had to be under the influence of illegal drugs or taking medication affecting body weight.

Inclusion criteria for the study included: (a) having participated in the relevant sport for at least 1 year at an elite level; (b) not having suffered injuries that caused them to miss more than three training sessions in the last 30 days before the study; and (c) completing the data collection sessions.

### 2.2 Ethics statement

All participants were verbally informed of the purpose of the study and gave written informed consent before testing. The study conformed to the ethical guidelines described in the Declaration of Helsinki (Anon, 2013) and received approval from the Ethics Committee for Research Involving Human Subjects of the University of the Basque Country, Spain under the number: M10_2017_216. The research was conducted in compliance with Organic Law 15/1999, of 13 December, on the Protection of Personal Data.

### 2.3 Experimental protocol

**FIGURE 1:**
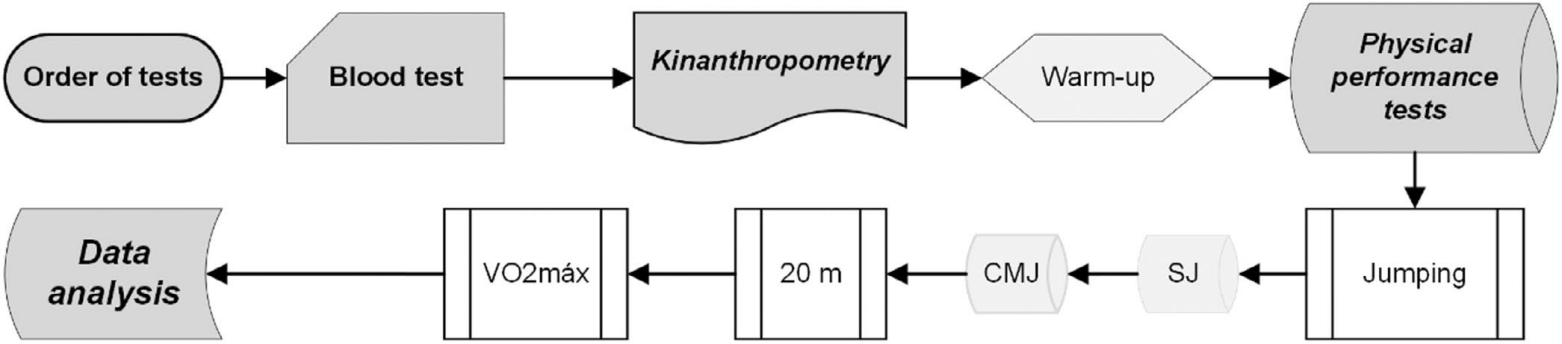
Experimental protocol.

The duration of the transition period was set at 16 weeks. All players were assessed on two unique occasions. Each experimental test consisted of 1 day of consecutive measurements (Figure 1). The first experimental test took place before the competition period (September 4 and 17). The second experimental test took place at the first competition stop (January 3 and 7). In each experimental period, testing consisted of blood tests to assess vD levels, anthropometric measurements (height, body weight, percentages of fat and lean mass), and ergometry tests to measure SJ (cm), CMJ (cm), 20 m sprint, and VO_2max_ (mL/kg/min). On the day of each experimental period, at 09:00 am, blood samples were obtained to determine the concentration of vD, then the anthropometric characteristics of each of the participants were measured, and in the afternoon of the same day (17,00) the players were subjected to SJ, CMJ and 20 m sprint tests and VO_2max_ was obtained indirectly with the IET Yoyo test. Before the tests, a 20‐min warm‐up was carried out (Verhagen et al., 2023). A 5‐min break was taken between each attempt (Lopez‐Samanes et al., 2021). Exercises were selected based on performance efficiency in each of the disciplines. Each test was detailed to the athletes (Lopez‐Samanes et al., 2021).

The measurements were taken in the sports hall where the players train and compete in the same session, to prevent variations in environmental or biological conditions from affecting the results (Halson, 2014). In volleyball: pavilion “El Ferial”—Haro (La Rioja) (T1: humidity 42%; temperature 28.1°C. T2: humidity 39%; temperature 14.9°C). 3% humidity difference and 13.2°C temperature difference between times. In basketball: municipal sports centre “José Antonio Gasca”—San Sebastián (Guipúzcoa) (T1: humidity 71%; 28.7°C. T2: humidity 47%; 16.4°C). 24% difference in humidity and 12.3°C difference in temperature between both moments.

For the evaluation, pretest actions were controlled to ensure that no physical exercise was performed in the 24 h before the analysis. In the 4 h before the test, no solid or liquid food was to be consumed, only a correct state of hydration and urination and/or defecation in the last 30 min before data collection (Liska et al., 2019).

### 2.4 Anthropometric measurements and body composition

Height measurement was decided using the Holtain® stadiometer (Holtain Ltd., Dyfed, UK) with millimeter accuracy. The measuring range is from 60 to 209 cm. Weight was obtained using an electronic scale with a SECA® scale (Seca Corp., Hanover, Md., USA) (accuracy: 0.1 kg; range: 2–130 kg). BMI was calculated using the formula BM/height^2^ (kg/m^2^). Body fat percentage was assessed by skinfold measurements taken in triplicate using a Harpenden® skinfold caliper and analyzed by two observers. The sum of 8 skinfold measurements (mm) (biceps, triceps, subscapular, iliac crest, supraspinal, abdominal, anterior thigh, and calf) was calculated and the equation developed by Carter & Heath (Heath & of Pennsylvania) Honeyman, 1990 was used to determine the body fat percentage of the different somatotypes (mesomorphy, ectomorphy and endomorphy) in women % fat mass = 3. 5803 + (Σ8 skinfolds*0.1548).

### 2.5 Vitamin D measurement

Blood samples were taken after a 10‐min rest in a lying position, following an overnight fast. The vD levels were obtained using the Vacutainer vacuum system with anticoagulant. Concentrations of calibrators 1 and 2 (human serum) refer to standard preparations holding highly purified 25(OH)vD. According to the manufacturer, the correlation of the immunoassay with high‐performance liquid chromatography (LC) coupled to mass spectrometry (MS) is described by the equation: concentration = 5.6 + 0.83*LC–MS/MS (*r* = 0.93). The intra‐assay coefficient of variation ranges between 3% and 6%. In our study, vD levels below 20 ng/mL (50 nmol/L) [3] were considered insufficiency. A concentration of 21–29 ng/mL (52–72 nmol/L) can be considered a relative deficiency of vD, while a value equal to or higher than 30 ng/mL can be considered a sufficient concentration (Ip et al., 2022).

### 2.6 Jump tests/sprinting ability

The jumping ability (SJ, CMJ) was measured using a Chronojump® Bosco System DIN‐A1 contact platform (De Blas et al., 2012), which has a microcontroller with a validity of 0.95 in the ICC, with an error of 0.1%. The mean error for the low signals (corresponding to the contact time in a jump) was 0.04 ± 0.18%, while for the high signals (corresponding to the flight time) it was 0.05 ± 0.19%. Also, the sprint capacity (20 m) was measured using MicroGate® Witty Wireless Timing Systems S.R.L. (Bolzano, Italy) photocells following standard procedures (Petway et al., 2020; Rodríguez‐Rosell et al., 2017; Markovic et al., 2004; Tenelsen et al., 2019; Chow et al., 2022; Altmann et al., 2019). These cells have an event delay of 1 millisecond and a resolution of 0.125 milliseconds. Self‐correction and accuracy checking are also available.

### 2.7 Ergometry endurance tests

Maximal oxygen uptake (VO_2max_) was measured indirectly with the IET yo‐yo test following the established procedures of a standard protocol (Martínez‐Lagunas & Hartmann, 2014). For the endurance test, the Yo‐yo Pro4.49® application was used, an application for group testing and advanced individual testing to perform the intermittent yo‐yo test (Recovery Level 1, 2 and Endurance Level 1, 2). Thus, the literature recommends the use of this test to measure the ability to repeat high‐intensity intermittent efforts and/or the recovery ability of this type of exercise. Therefore, its validity and applicability have been studied in several team sports (García & Secchi, 2013). The validity of this test is based on the association obtained between the metres accumulated in the test and the total performance in competition (total metres run) and/or the metres run at high intensity (runs above 15 km/h).

### 2.8 Reaction speed and acceleration

Participants could perform two 20 m sprint attempts from a stationary position, recording the fastest attempt to extrapolate the data from this test to both the sample (internal validity) and the target population (external validity) and not performing further attempts as speed should be performed without fatigue with very long rest pauses that would interfere with the ecological validity of the measurement, as although repeated attempts often lead to improved accuracy, these concepts are distinct and unrelated (accuracy is possible without precision, and vice versa) (Andrade, 2018). Witty‐Gate photocells manufactured by MicroGate® Timing Systems S.R.L. in Bolzano (Italy) were used to record the start and finish line of the sprint.

The 20‐metre speed test, which has demonstrated intra‐class correlation indices of 0.11–0.49 and coefficients of variation of 16.8%–51.0%, the test–retest correlation coefficient of 0.91 indicating high levels of reliability (without the need for pretesting) (Altmann et al., 2019).

### 2.9 Statistical analysis

Results are presented as mean ± standard deviation. The distribution of variables was assessed using the Shapiro–Wilk statistical method (<30). The percentage change (Δ%) from T1 to T2 in outcome variables was calculated using the following formula: [(T2‐T1) /T1] × 100. With a sample of 23 participants, G*Power 3.1.9.7 software was used to determine the sample size (22.54), power analysis and effect size. Partial eta squared (*η* ^2^_p_) was calculated as a measure of the degree of influence between individuals. This metric tends to overestimate the impact, so it was interpreted as follows: *η* ^2^_p_ values between 0 and 0.05 say no effect, between 0.05 and 0.26 write down a small effect, between 0.26 and 0.64 write down a moderate effect and values equal to or greater than 0.64 show a large effect. Reference is also made to estimated probabilistic inference (Batterham & Hopkins, 2006). Next, Pearson correlation coefficients (for normally distributed variables) and Spearman correlation coefficients (for non‐normally distributed variables) were used to assess the linear relationship between quantitative variables. Correlation coefficients were determined as trivial (*r* < 0.1), small (0.1–0.3), moderate (0.3–0.5), high (0.5–0.7), very high (0.8–0.9), near perfect (0.8–0.9), and perfect (*r* = 1) (Hopkins et al., 2009). Changes between experimental periods in measured parameters within groups were analyzed using the paired *t*‐test for normally distributed data and the Mann–Whitney test for non‐normally distributed data. Statistical analysis was performed with SPSS 25.0® software (Inc., Chicago, IL, USA). The significance level was set at *p* < 0.05.

## 3 RESULTS

### 3.1 Changes in vD levels, exercise performance parameters, and body composition

**FIGURE 2:**
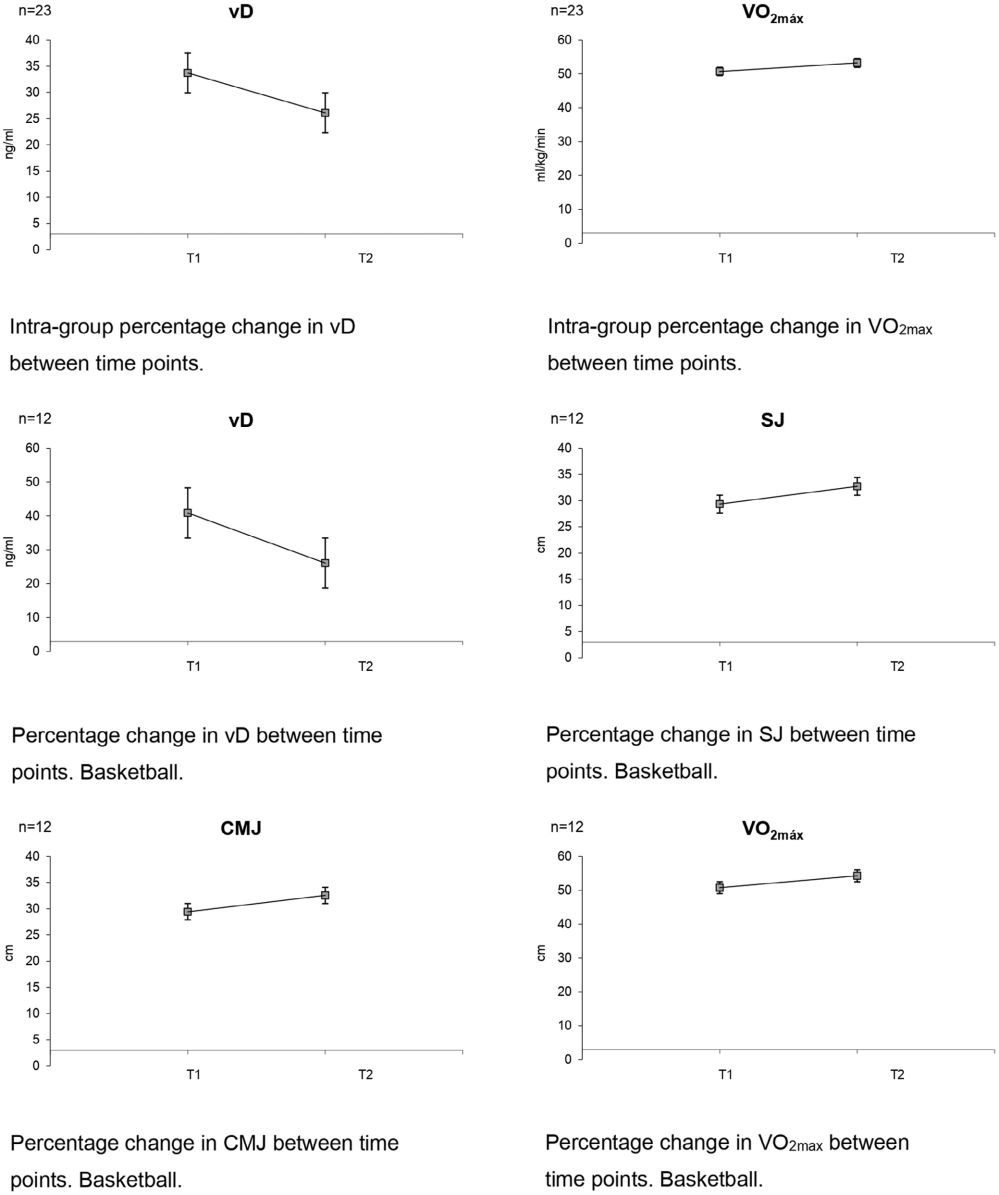
High (>10%) or significant percentage changes.

At the intragroup level, the vD values and exercise performance parameters at baseline and after the 16‐week transition period are presented in Table 1. The vD levels decreased at the second data collection time significantly compared to the baseline values (Table 1). In contrast, analysis of our data revealed an increase (Figure 2) in the values of all jumps and VO_2max_, although only the change in VO_2max_ levels is shown to be significant at the second measurement time compared to baseline values (Table 1). At the intergroup level, the volleyball group shows an increase in vD levels. Regarding jumping the data are contradictory showing a decrease in one (SJ) and an increase in the other (CMJ). As well as improvements in VO_2max_, but all these changes are not significant. In the other group (basketball; *n* = 12) the same trend occurs at the intragroup level with a decrease in vD levels and an increase in sports performance marks, with only the change in vD being significant.

**TABLE 1:** Correlations (correlation coefficients and *p* values) between vD levels and exercise performance parameters.

Performance testing	T1 vD (ng/mL)	T2 vD (ng/mL)
(*n* = 23)		
SJ (cm)	0.333	−0.075
CMJ (cm)	0.012	−0.515
VO_2max_ (mL/kg/min)	−0.106	0.021
20 m (s)	−0.016	0.454
(*n* = 11)		
SJ (cm)	−0.549	0.546
CMJ (cm)	−0.475	−0.505
VO_2max_ (mL/kg/min)	−0.308	0.350
20 m (s)	0.293	0.333
(*n* = 12)		
SJ (cm)	0.382	−0.367
CMJ (cm)	0.247	−0.554
VO_2max_ (mL/kg/min)	−0.094	−0.344
20 m (s)	−0.025	0.641

Abbreviations: CMJ: counter movement jump; SJ: squat jump; T1: time 1; T2: time 2; vD: vitamin D; VO_2max_: maximum oxygen consumption.

Finally, a decrease in body weight (74.02 ± 11.50 vs. 73.03 ± 10.51; *p* < 0.05), % fat mass (18.5% ± 4.0 vs. 18.1% ± 3.4; *p* > 0.05), and % fat‐free mass (34.1% ± 3.4 vs. 35.2% ± 3.3; *p* > 0.05) was evident at the end of the study compared to the initial intragroup value. At the intergroup level, in group *n =* 11 (volleyball) fat mass % was kept (17.3%) and fat‐free mass % increased (34.2% ± 3.7 vs. 35.7% ± 3.9; *p* > 0.05). In group *n* = 12 (basketball), there was a decrease in fat mass (19.7% ± 4.4 vs. 18.8% ± 3.2; *p* > 0.05) and an increase in fat‐free mass (33.9% ± 3.2 vs. 34.7% ± 2.7; *p* > 0.05).

### 3.2 Correlation between vD levels and exercise performance parameters

**FIGURE 3:**
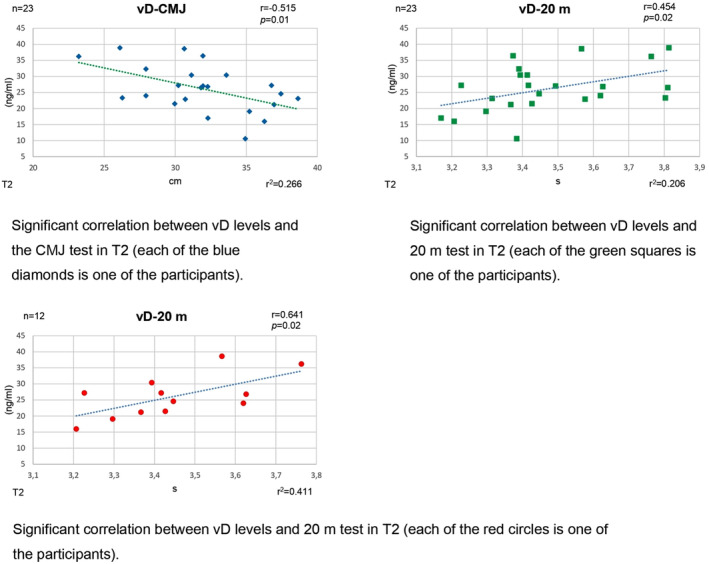
Significant correlations.

Table 2 shows the correlations (Figure 3) between vD levels and exercise at the beginning and end of the 16 weeks. Our analysis revealed a moderate‐to‐high correlation at the intragroup level between vD levels and CMJ values (*r* = −0.515 [high]; *p =* 0.01 [significant]) and as with 20 m (*r* = 0.454 [medium]; *p* = 0.02; *p =* 0.02 [s]) at T2 (Table 2). At the intergroup level (volleyball; *n* = 11), we can observe a moderate or high level of correlation between vD and SJ (*r* = −0.549 [h]; *p =* 0.08 [ns]), CMJ (*r* = −0.475; *p =* 0.01 [s]), and VO_2max_ (*r* = −0.515 [h]; *p =* 0.01 [s]) values at T1 and also a moderate or high level of correlation between vD and all sport performance outcomes. In the other group (basketball; *n* = 12), a moderate correlation of vD parameters with SJ jump scores is seen at T1. At T2, moderate‐to‐high correlations are seen between DV levels and all sports performance outcomes.

**TABLE 2:** Vitamin D values and yield in the two experimental periods.

Measures	T1	T2	*t*	*η* ^2^_p_	%Δ	*p*	Change magnitude	Probabilistic inference
(*n* = 23)								
Vitamin D (ng/mL)	33.7 ± 14.7	26.1 ± 7.3	2.036	0.650	−22.40%	0.03	Strong	Very likely harmful
SJ (cm)	27.2 ± 4.1	29.0 ± 5.5	2.019	−0.260	4.57%	0.38	Minimal	Possibly beneficial
CMJ (cm)	29.9 ± 4.4	31.9 ± 4.0	2.015	−0.493	6.94%	0.1	Moderate	Possibly beneficial
VO_2max_ (mL/kg/min)	50.7 ± 4.1	53.2 ± 3.5	2.016	−0.665	4.99%	0.02	Strong	Possibly beneficial
20 m (s)	3.5 ± 0.2	3.5 ± 0.2	2.016	0.316	−1.83%	0.28	Moderate	Possibly trivial
(*n* = 11)								
Vitamin D (ng/mL)	25.8 ± 9.1	26.2 ± 8.2	2.086	−0.045	1.51%	0.92	No effect	Possibly trivial
SJ (cm)	26.0 ± 2.9	25.0 ± 1.8	2.110	0.436	−3.98%	0.32	Moderate	Possibly harmful
CMJ (cm)	30.3 ± 3.2	31.3 3.6	2.086	−0.277	3.12%	0.52	Moderate	Possibly beneficial
VO_2max_ (mL/kg/min)	50.6 ± 2.6	52.1 ± 3.4	2.093	−0.501	2.98%	0.25	Moderate	Possibly beneficial
20 m (s)	3.6 ± 0.2	3.5 ± 0.2	2.086	0.322	−1.92%	0.45	Moderate	Likely harmful
(*n* = 12)								
Vitamin D (ng/mL)	40.9 ± 15.5	26.1 ± 6.6	2.131	1.243	−36.24%	0.008	Strong	Very likely harmful
SJ (cm)	29.3 ± 4.5	32.7 ± 5.2	2.073	−0.693	11.52%	0.1	Strong	Probably beneficial
CMJ (cm)	29.4 ± 5.4	32.5 ± 4.3	2.079	−0.633	10.54%	0.1	Moderate	Probably beneficial
VO_2max_ (mL/kg/min)	50.8 ± 5.3	54.2 ± 3.4	2.093	−0.784	6.83%	0.06	Strong	Possibly beneficial
20 m (s)	3.5 ± 0.2	3.4 ± 0.2	2.085	0.303	−1.74%	0.46	Moderate	Possibly trivial

Abbreviations: CMJ: counter movement jump; n2p: Partial eta squared; %Δ: percentage of change; p: significance level; SJ: squat jump; T1: time 1; T2: time 2; t: t‐student test; VO_2max_: maximum oxygen consumption.

## 4 DISCUSSION

This study aimed to see the evolution of vD during the 16‐week transition period of the season and its relationship with performance tests in a group of 23 elite female indoor sports athletes. Data are taken at two points in the season in which the absolute and percentage changes of the parameters to be seen can be seen (Table 1).

We hypothesized that vD levels would be related to jumping (SJ and CMJ), sprinting (20 m sprint without change of direction), and participants' aerobic capacity (VO_2_ max). Furthermore, the transition between the two moments improved vD concentration and, therefore, athletic performance. Our results showed that vD levels are associated with the neuromuscular pattern of jumping (SJ and CMJ) and the aerobic ability of PFISPs. Importantly, the study provides evidence of a linear relationship between serum vD levels, not only with jumping performance but also with VO_2_ max and speed in professional female athletes in indoor team sports without the use of any supplementation. Similarly, we have curiously observed that, at a general level, the decrease in vD levels was evident in parallel to a significant improvement in aerobic and neuromuscular performance (vD: −22.4%; SJ: 4.57%; CMJ: 6.94%; VO_2max_: 4.99%; 20 m: −1.83%), not significant in the latter case. This latter finding reinforces the previously well‐documented concept that training plays a major role in exercise adaptation and performance enhancement, while parameters such as vD correlate with various aspects of physical performance, as it has been observed that deficient levels of 25‐hydroxyvitamin D [25(OH)D] (<30 ng/mL) can compromise health and athletic performance (Mielgo‐Ayuso et al., 2018).

In recent years, sports medicine experts have begun to study how vD affects athletes, especially those involved in high‐intensity sports (Mielgo‐Ayuso et al., 2018) and female athletes (Agostini et al., 2018). These studies (Farrokhyar et al., 2015) have revealed that many athletes, especially those who play indoor sports, are deficient in vD, that is, they have vD levels below the recommended 25(OH)D < 30 ng/mL (Şenışık et al., 2022). This can have negative consequences for both their health and athletic performance (Most et al., 2021). Maintaining adequate levels of vD is advised, as it improves muscle protein production, increases energy levels, strengthens muscles, improves speed and power, and increases performance in both aerobic and anaerobic exercise (Yagüe et al., 2020; Agostini & Donati Zeppa, 2023).

### 4.1 Jump

vD levels showed a positive linear relationship with muscle strength (SJ and CMJ) at T1 and negative at T2 as DV levels decreased. In the case of the evaluation, we can consider that this correlation is related to the performance results obtained in that vD levels have decreased between times, but performance improves both intragroup (*n* = 23) and intergroup, probably caused by the training that has an impact on the increase in muscle mass (Bernárdez‐Vázquez et al., 2022) and in turn on the increase in blood testosterone (T) levels (Riachy et al., 2020). Furthermore, the same is true for the results of the speed test, where the % changes are seen to decrease, indicating a slight improvement in test performance times, perhaps influenced by the same reasons for the influence of blood T concentration on the results of explosive performance tests (Cardinale & Stone, 2006).

It is worth mentioning that both the SJ and CMJ are considered standard goal field tests (Marcovic et al., 2004), to assess lower extremity strength levels (Petrigna et al., 2019), already published in a multitude of previous studies in basketball (Ramirez‐Campillo et al., 2022) and volleyball (Berriel et al., 2021).

### 4.2 VO_2max_

Analysis of our data revealed a moderate linear association between vD and VO_2max_ at T2 at the intergroup level. This finding can be supported by a previous study justifying aerobic capacity as a result of exposure to ultraviolet radiation (Koundourakis et al., 2014). In addition, another study observed a positive relationship between vD and aerobic performance (Brzeziański et al., 2022). Given that VO_2max_ is related to performance both in volleyball, as aerobic power is an important factor in this discipline for good recovery between points, sets, and sets of matches, and in basketball (Escribano‐Ott et al., 2022), as it allows better recovery from anaerobic efforts (Mielgo‐Ayuso et al., 2018) and, therefore, a delay in the onset of fatigue mainly from a succession of anaerobic efforts, achieving better assimilation and maintenance of technical efficiency for longer (Poole et al., 2016). Our findings suggest that optimal levels of vD are required for performance during a match in either of these two indoor modalities (Yagüe et al., 2020). The observed association between vD levels and VO_2max_ may in turn be related to its protective effect on lung function (Hassel et al., 2015). According to recently published evidence, low vD levels are associated with lower rates of lung function (Wang et al., 2022) and increased airway reactivity (Gaudet et al., 2022). Since exercise performance and especially aerobic capacity (VO_2max_) is dependent on lung function (Hackett, 2020), any protective and/or potentiating effect of vD on lung function could beneficially influence aerobic performance during exercise (Patel et al., 2017). vD may also influence VO_2max_ by affecting iron and erythropoietin metabolism (Shoemaker et al., 2022). There is evidence that vD deficiency leads to dysregulation of innate immunity and inflammation that affects iron metabolism and contributes to erythropoietin resistance (Uwaezuoke, 2017). It is also well‐documented that erythropoietin is linearly associated with changes in red blood cell levels (Weiss et al., 2020). Thus, vD could influence VO_2max_ through its effects on erythropoiesis by modifying the oxygen delivery capacity of exercising muscles and, consequently, affecting aerobic exercise performance (Most et al., 2021).

### 4.3 Translational velocity

In this study, it has been observed from the results that the involved lower limb musculature is affected by vD deficiency (Green et al., 2022). Aspects that affect the translation velocity (20 m WCD) in T2 showing a positive, moderate, and significant. So based on these indications, our findings suggest a possible effect of vD on power and strength, which in turn translates into sprint performance as vD induces the process of muscle tissue cell determination and formation and muscle protein synthesis, leading to an increase in the percentage of fast twitch muscle cells (type II fibers) (Wiciński et al., 2019).

The pathways by which blood levels of vD affect muscle strength and sprint performance are still hypothetical to our knowledge. However, there are several mechanisms that may potentially be responsible to some extent for such effects. For example, the ergogenic effects of vD may be related to the regulation of muscle protein synthesis, which could affect muscle mass, due to the presence of vD receptors on skeletal myocytes (Ksiażek et al., 2019). In addition, alterations in vD levels also affect its receptors at the level of expression and activation and thus muscle mass, neuromuscular coordination and the relative number and cross‐sectional area of type II muscle fibers (Bikle, 2016). Given that it is well documented that the main determinants of jumping and sprinting ability are muscle strength, type II muscle fibers, and neuromuscular coordination (Methenitis et al., 2016), any potential effect of vD on these parameters would affect sprinting ability, and similarly to jumping as seen above (Bashir et al., 2022). Future studies should carefully explore the effects of vitamin D supplementation on strength and speed performance in elite female athletes in these disciplines. In addition, it would be important to quantify how several types of training and exercise stress affect vitamin D levels. It is possible that reaching a certain threshold serum 25(OH)D concentration is associated with changes in different measures of physical performance. It would also be valuable to assess the dynamics of serum 25(OH)D levels during the competitive season and to develop interventions to correct any detected deficiencies or insufficiencies. Finally, we believe that it is worth investigating the levels of this vitamin in athletes, considering the particularities of their training and competition activities.

### 4.4 Changes between time points

Interestingly, analysis of our data also showed that, although vD decreased between the two periods, all measured performance parameters increased. However, the linear correlation observed at baseline between vD levels and performance was greater at T2, suggesting that it is not clear that maintaining theoretically correct vD levels is related to the ability to perform effectively during exercise, regardless of performance level (Angulo et al., 2020). These data strongly suggest that, although vD appears to affect neuromuscular and aerobic performance, it does not play a major role, demonstrating that the main determinant of exercise performance is the quantity and quality of training (Yagüe et al., 2020). This latest evidence documents that vD plays a supportive role in exercise performance but does not detract from the importance of serum vD concentration, as, at the elite sport level, slight changes in these levels can figure out performance levels. However, this process does not diminish the importance of serum vD concentration, as in these elite‐level disciplines even subtle changes in performance can determine the outcome of a competition (Nemkov et al., 2023).

In this study, the level of vD insufficiency detected was lower than in previous studies in both athletes and the general population (below 20 ng/mL) (Bezuglov et al., 2023). While a systematic review indicated that more than 37% of the world's population has circulating 25(OH)D concentrations below 20 ng/mL (Bezuglov et al., 2023). Studies on athletes have also confirmed the high prevalence of serum vD deficiency, showing that 64%–84% of athletes tested were deficient (Ip et al., 2022). Future studies should analyze and compare such biomarkers with the external load of training and matches (e.g., by accelerometers, GPS, or movement time counting), as the sports population has special characteristics. Also, explore other relationships such as psychological state (e.g., through questionnaires such as POMS, RESTQ‐Sport, or STAI). On the contrary, nutritional parameters and sleep quality could supply valuable information on load and fatigue throughout the season.

In our study, the 16‐week transition period had a negative effect on vD levels at the intragroup level. Indeed, in the first experimental period, 13% of the participants were deficient in vD (<20 ng/mL), all volleyball players, and 39.1% of our players had insufficient vD levels (<30 ng/mL) (21. 7% volleyball; 17.4% basketball), while in the second the percentage of players with deficits increased to 17.4% (8.7% in both disciplines), and 52.2% had levels below 30 ng/mL (21.7 volleyball; 30.5% basketball). Thus, with mean concentration values approaching the lower limit of normal, possibly suggesting the need to monitor the vD biochemical marker 25(OH)D during the autumn and winter months (Bezuglov et al., 2023), when the level of sun exposure is significantly reduced, and factors possibly affecting vD status include exercise stress and exercise itself. In a study (Andersen et al., 2010) investigating a female team, it was shown that exercise stress can play a role in regulating vD levels and reducing vD concentrations even during prolonged outdoor exercise in the summer and early autumn months (Andersen et al., 2010). Thus, exercise‐induced stress may affect vD status in athletes, possibly related to the immunosuppressive effect of intense stress (Crescioli, 2022). Exercise itself, however, may also increase vD levels. This has been found in some observational studies, which showed that regular exercise or high physical activity was associated with higher serum 25(OH)D levels, even after adjusting for sun exposure (Sun et al., 2017). One of the major contributing factors to vD deficiency in athletes is residence in northern regions due to the greater angle at which the sun's rays enter the atmosphere in these regions, resulting in scattering (Ip et al., 2022). This concept is supported by research examining athletes living at 60° north latitude, which found deficiencies in serum 25(OH)D concentration in more than 80% of this subject population (Bezuglov et al., 2023). Also a descriptive review (Ksiażek et al., 2019) showed that previous studies were inconclusive and found no clear association between serum 25(OH)D concentration and performance. Therefore, it can be argued that the available data on the effect of vD on physical performance are inconsistent and limited to assessing the effect of supplemental vD intake on muscle function in athletes with vD deficiency or insufficiency. Studies investigating high‐level athletes and the effect of actual vD status on muscle function are currently scarce to our knowledge. Although there are studies that have obtained significant correlations between vD status and SJ, CMJ, and performance in sprints such as the 20 m (Contreras‐Bolívar et al., 2021; Książek et al., 2022; Voltan et al., 2023).

The most plausible explanation for the decrease in vD levels in the second session could be a consequence of lower ultraviolet B (UVB) exposure during the chosen winter period of the season (Hamilton et al., 2014), as the extreme importance of UVB in the synthesis of vD is well demonstrated (Veleva et al., 2020) and the observation that its efficacy, among other parameters, depends on the season (Hays et al., 2022), as due to the geographical location, the daylight hours in the north of the country, both disciplines train under cover in closed sports centres (Şenışık et al., 2022).

The data presented here reaffirm the importance of adequate UVB exposure, which is supported by recent evidence that exercise may play a role in regulating vD levels (Raymond‐Lezman & Riskin, 2023). This calls for close monitoring of this variable in such indoor sports, especially in northern latitude countries.

Future studies should investigate aspects such as changes in vD levels throughout the competitive season, the effects of vD supplementation on strength and speed performance in elite female athletes by introducing a control group, quantifying the effect of various types of training and exercise stress on vD status, or examining the possible association between vD supplementation and elevated serum 25(OH)D concentration and the effect on total T concentration.

## 5 CONCLUSIONS

Our study supplies evidence of an association between non‐supplemented vD levels and aerobic and neuromuscular exercise performance parameters in female indoor disciplines. Our data show a linear relationship between vD levels and muscle strength assessed by SJ and CMJ, sprinting ability (20 m), and VO_2max_ in PFISPs. Furthermore, our results write down that, apart from increased UVB exposure, increased training stress may also have detrimental effects on vD levels in elite indoor female players. However, further research that also examines inflammation indices is needed to confirm this hypothesis. Furthermore, our results show that vD plays, among other parameters, a secondary supportive role in sports performance. However, this does not diminish its importance, especially for elite female athletes, as slight changes in performance can figure out the outcome of a competition as well as general health levels.

## 6 STRENGTHS AND WEAKNESSES

Our main strength is to have evaluated the complete endocrine and anthropometric profiles, with blood samples collected following a standardized protocol until their analysis. And although we have tried to always support the ecological validity of the process without intervening during events, the fact of being able to conduct the measurements in number and manner in elite sports teams are difficult factors to bear in mind in this type of work. On the one hand, the presence of the researchers and the measurement procedures can have an impact, however small, on the normal development of the activity and the behavior of the athletes. This poses a challenge in terms of keeping ecological validity, that is, that the study conditions are as close as possible to the everyday reality of the team. In addition, access to these elite teams is often highly restricted and controlled, making it difficult to collect data in the desired quantity and frequency, as coaches and managers tend to protect their athletes from anything that might distract them or disrupt their preparation routine, thus limiting research opportunities.

On the other hand, the important level of performance and the physical and mental demands to which these athletes are subjected require exceptional care and sensitivity on the part of the research team. Any intervention, however minor, can have a significant impact on their performance and well‐being.

So, while having elite sports teams as subjects of study offers a valuable opportunity to understand high‐interest phenomena, it also poses several methodological and ethical challenges that must be approached with great rigor and diligence by researchers.

Our study follows high‐level athletes for 16 weeks before and during a championship, using an integrated approach to examine physiological and performance variables. It highlights the opportunity of multivariate adjusted logistic regression analysis because the athletes assessed are young and healthy.

The generalizability of the results is limited by the scope and controlled conditions of the study, as well as the lack of a control group. The absence of data on sleep, diet, clear international threshold values for diagnosing deficiencies, and information on outdoor time are also limitations.

## 7 FUTURE RESEARCH LINES

Future studies should examine how vD supplementation affects strength and speed performance in elite female athletes. It would also be important to quantify how diverse types of training and exercise stress influence vD levels. It is possible that serum 25(OH)D concentrations above a certain threshold may be associated with changes in key measures of physical performance. In addition, it would be valuable to assess changes in serum 25(OH)D levels during the competitive season and to develop interventions to correct any detected deficiencies or insufficiencies. It is also worth investigating 25(OH)D levels in female athletes and considering the particularities of their training and competitive activities. Finally, exploring the possible relationship between vD supplementation, elevated serum 25(OH)D levels and the effect on catabolic hormones could also be an interesting topic for future research.

## 8 PRACTICAL APPLICATIONS

A thorough understanding of the internal effects that each athlete experiences during a competitive season can supply the opportunity to improve training strategies on an individual basis. It is recommended to monitor vD and hormone levels (via T and Cortisol) to prevent excessive stress caused by the demands of the professional sport season.

The findings highlight the importance of continuous monitoring of training load levels and biomarkers in female athletes to reveal the cumulative effects of stressors both in and out of competition. Monitoring biomarkers and training load together supplies a more complete picture of athletes' preparation and general health status, thus easing better management of athletes. Periodic assessments supply multiple opportunities to implement interventions that can prevent or mitigate performance declines, as seen in this study. Potential supplementation with iron, fatty acids such as omega‐3 and vD could be beneficial for many female athletes to support reliable performance throughout the season.

## AUTHOR CONTRIBUTIONS

Conception and design of experiments, writing—review and editing, and visualization: ÁM‐O and JC‐G; methodology, formal analysis the data, and investigation: ÁM‐O, JC‐G, and JM‐A; software, writing—original draft preparation, and project administration: ÁM‐O; supervision: JC‐G and JM‐A. All authors have read and agreed to the published version of the manuscript.

## FUNDING INFORMATION

The authors declare that they have no financial or non‐financial interests in any company that supports their research, takes part in discussions on the topic or supplies materials for this study.

## CONFLICT OF INTEREST STATEMENT

The authors declare that they have no conflicts of interest.

## ETHICS STATEMENT

The basis for this study was the World Medical Association Declaration of Helsinki (2014) on medical research involving human subjects. In addition, the research was approved by the Human Research Ethics Committee of the University of the Basque Country (number M10_2017_216). It is important to note that the data were collected with the utmost discretion and scientific integrity. Organic Law 15/1999 of 13 December 1999 on the Protection of Personal Data (LOPD) requires that research projects follow a scientific method. The law 14/2007, which can be found in issue 159 of the OSG, set up ethical standards for the methods used.

## INFORMED CONSENT

All study participants gave informed consent. Data availability statement: The article holds the information.

## Data Availability

To increase the transparency of the data supporting the conclusions reported in the article, the authors have implemented a data availability statement. The data linked to this article are not publicly available but can be requested from the corresponding author.
